# Editorial Department

**Published:** 1886-07

**Authors:** 


### EDITORIAL DEPARTMENT.

Dr. Spitzka on Hydrophobia.—We devote a large space of our journal to Dr. Spitzka's able paper on spurious hydrophobia, together with the discussion thereon. The doctor shows that he can produce the same disease in a dog by inoculating almost any foreign substance into the brain, as Dr. Liautard obtained by inoculation from a supposed rabid dog. The author of the paper seems to have been somewhat misunderstood, if we are to judge from recent editorial notices in the daily journals. He did not, as they assume, or as one of the speakers of the evening assumed, claim to have produced genuine hydrophobia, or deny the existence of such a disease. He distinctly repudiated discussing that question, and it appears to have been his main purpose to quiet public alarm by showing that genuine rabies is an extremely rare affection and that at present it cannot be pronounced to be epidemic either in New York or its vicinity. Regarding it from a purely philanthropic standpoint, Dr. Spitzka's observations should be widely circulated. They point out the influence of fear in stimulating the symptoms of hydrophobia, and even inducing the disease itself. In view of these facts, and the special pains taken—as we are informed—to secure the presence of the incorporators of the Pasteur Institute of this city at the reading of the paper, and eliciting their views, we cannot but regard it as most unfortunate that both Drs. Mott and Liautard were compelled to excuse themselves owing to other engagements. It is not calculated to create a pleasant impression that no representative of an institution which is calculated to flourish by appeals to public confidence, saw fit to be present at so important a meeting as the one which took place last Thursday. One of the most well-timed and thoughtful suggestions made in the course of the discussion was that made by Dr. Dulles, of Philadelphia. Dr. Dulles is an outspoken opponent of the existence of lyssa in man, in the sense that term is used in most text-books and treatises. He called attention to the fact, and we are surprised it has not been touched on before, that Pasteur obtained his original virus (with whose derivatives he is now working) from a *single dog*, as to whose having had rabies in reality there is great doubt, for Pasteur was at the time not an expert on rabies. It strikes us that, in view of this revelation, the suggestion of the *Deutsche Medizinische Wochenschrift* quoted in the paper read, as to the need for corroborative experiments is very well timed. A very important admission was frankly made by Dr. Biggs, of the Carnegie Laboratory. He confessed that though Neall, the Newark pound-keeper, was regarded as a subject of hydrophobia, inoculations of rabbits and dogs, made by himself and by Prof. Law, of Cornell, proved entirely negative. Although he took opposite ground to Drs. Dulles and Brill—the latter of whom delivered a telling indictment of Pasteur's peculiar announcement *apropos* of German opposition, he really sustained their position against Pasteur, by admitting that the rabbit has no characteristic signs of rabies. The importance of this admission is due to the fact that Pasteur, for some unaccountable reason, has not selected the dog, but the rabbit, as the medium for the cultures of hydrophobia virus. Dr. Bigg's admission also invalidates the value of the experiments made with material from Dr. Kretchmar's Brooklyn patient, on a rabbit by Dr. Sternberg, in Baltimore. The paraplegia found in the latter case was pronounced by Dr. Biggs exactly as that in Dr. Liautard's case was stigmatized by Dr. Spitzka as the result of ordinary brain paralysis. C.

---

Ozone and Pneumonia.—Dr. Daniel Draper, Director of the Central Park Meteorological Observatory, in his report for the year 1885, furnishes some very interesting data on the subject of pneumonia. Having made a systematic study of this disease from a meteorological standpoint, comparing the mortality statistics obtained from the Board of Health, with his recorded observations, he found that a high death rate from pneumonia corresponded with an increase in the quantity of ozone in the atmosphere, and *vice versa*. The connection between these conditions, although not sufficient to demonstrate their relation of cause and effect, justifies further investigation. We would suggest to those in charge of large numbers of animals to keep a record of the cases of pneumonia occurring amongst them, and make a report of the same. Veterinarians would then be enabled to make comparisons between these cases and the variations in the quantity of ozone in the atmosphere, obtainable at the nearest meteorological station, and thus help to throw light on this important subject. To establish the fact that there existed a connection between ozone and *cases* of pneumonia would be of much greater value in the elucidation of the question than to demonstrate the relation between the same atmospheric conditions and the *deaths* from pneumonia.

C.

---

VETERINARY LEGISLATION.—The New York State Veterinary Society has succeeded in procuring the passage by the Legislature of this State of a bill to regulate the practice of veterinary medicine. The first section of the bill states that every practitioner must be duly registered in the office of the Clerk of the County in which he resides. The second section provides that no person is entitled to register unless he be a graduate of a legally chartered or incorporated college or university, or holds a certificate from a legally incorporated veterinary society. The third section admits all persons who have been practising veterinary medicine and surgery three years preceding the passage of this act, and who are without the necessary diploma or certificate, to register the same as those provided for in section two; provided, the same is done within six months from the passage of this act. The rest of the act provides that any person practising veterinary medicine and surgery without conforming to this act shall be guilty of misdemeanor, and liable to a fine of from $50 to $250 for each offence.

---

DIAGNOSIS OF TUBERCULOSIS IN CATTLE.*—Leidmann deduces the following conclusions from some practical studies upon this question: 1st. The mucous secretion of the trachea always contains the specific bacilli when extensive processes of that nature are seated in the lungs. The germs can always be diagnosed intra vitam, but failure in doing so would not neces-

* *Wochenschrijf fur Thierheilkunde*, No. 9, 1886.

sarily exclude the possible existence of tubercular processes in the lungs. 2nd. The milk of tuberculous cows contains the bacilli, even when no macroscopical evidences of the disease are observable in or around the udder. 3d. Tubercular processes in the intestinal canal can also be diagnosed by the presence of the specific bacilli in the discharges.

---

We beg to acknowledge the receipt of the kind invitation extended to us by the members of the Medical Press and Library Association in behalf of the "Weekly Medical Review," to attend at a dinner at the St. Louis Club, and to express our sincere regret at our being unable to be present at such an agreeable reunion.

